# An Overview of Tools to Score Severity in Pediatric Inflammatory Bowel Disease

**DOI:** 10.3389/fped.2021.615216

**Published:** 2021-04-12

**Authors:** Ron Shaoul, Andrew S. Day

**Affiliations:** ^1^Pediatric Gastroenterology & Nutrition Institute, Ruth Children's Hospital of Haifa, Rambam Medical Center, Faculty of Medicine, Technion, Haifa, Israel; ^2^Department of Paediatrics, University of Otago Christchurch, Christchurch, New Zealand

**Keywords:** children, inflammatory bowel disease, assessment, scoring, endoscopy, MRE, Crohn's disease, ulcerative colitis

## Abstract

**Background and Aims:** The management of IBD entails the use of various treatments (nutrition, medications, and surgery) in order to induce and maintain remission. The assessment of IBD disease activity is based on a combination of symptoms, clinical findings, imaging, and endoscopic procedures. As in any disease, reliable assessment of disease activity or severity is required in order to plan relevant follow-up, decide on appropriate investigations, determine the best treatment option and subsequently assess response to treatment. It is important for proper documentation, follow-up, assessment of response to treatment and communication, especially in patients with IBD, to talk the same language by using validated and widely used scores for disease activity, endoscopic and radiologic activity, and patient reported outcomes both for clinical practice and research. This review aims to highlight key tools available for the assessment of disease activity or severity in individuals (especially children) with IBD.

**Methods:** A literature search was performed using MEDLINE, Pubmed, and the Cochrane Library with the last search date of August 2020. Tools evaluating disease severity across various aspects (clinical, endoscopic, and radiological) were identified and discussed. Those tools validated and specific for children with IBD were included were available.

**Results:** Over time a number of scoring systems have been developed to quantify clinical, endoscopic and imaging assessments in individuals with IBD. While some are exclusively for children or adults, others appear to have relevance to all age groups. In addition, some tools developed in adult populations are utilized in children, but have not expressly been validated in this age group.

**Conclusions:** Although some available scoring tools are appropriate for children with IBD, others require consideration. The development and use of pediatric-specific tools is relevant and appropriate to optimal care of children and adolescents with IBD.

## Introduction

The inflammatory bowel diseases (IBD) are characterized by chronic inflammation of the gastrointestinal (GI) tract (1, 2). Typically, there are two main types: Crohn disease (CD) and ulcerative colitis (UC) (1, 2). Some individuals may have inconclusive features initially—the term IBD-unclassified (IBDU) is used in these circumstances.

The management of IBD entails the use of various treatments (nutrition, medications, and surgery) in order to induce and maintain remission. Mucosal healing is increasingly recognized as an important endpoint both in clinical practice and in the research setting (3). The assessment of IBD disease activity is based on a combination of symptoms, clinical findings, imaging and endoscopic assessment.

As in any disease, reliable assessment of disease activity or severity is required in order to plan relevant follow-up, decide on appropriate investigations, determine the best treatment option and subsequently assess response to treatment. The process of developing optimal clinical and research indices involves selection and refinement of test items, determination of reliability and validity, and examination of the performance of the measure in different settings and among different populations (4). These are the basis for most disease monitoring devices in clinical and research scenarios (4). This review aimed to highlight key tools available for the assessment of disease activity or severity in individuals with IBD, with a focus upon their applicability to children.

## Disease Activity Scores for Crohn's Disease

### Crohn's Disease Activity Index (CDAI) and the Harvey-Bradshaw Index in Adults With CD

The CDAI was the first clinical index for adults with CD (5). The National Cooperative Crohn's Disease Study group collected data prospectively from 187 visits of 112 patients with CD of the small bowel, colon, or both. Information on 18 predictor variables was gathered at each visit. In addition, the attending physician rated their over-all evaluation of how well the patient was doing and compared the patient's status with that at the previous visit. A multiple regression computer program was utilized to derive an equation for prediction of the physician's over-all ratings from a subset of the predictor variables fulfilling a combination of constraints. This equation, numerically simplified and utilizing eight selected variables, became the CDAI (5). Index values of 150 and below are associated with quiescent disease; values above that indicate active disease while values above 450 are seen with extremely severe disease (5). The CDAI has since been widely used in CD clinical trials.

The Harvey-Bradshaw Index (HBI) is a simplification of the CDAI, designed to make data collection and computation easier (6). It is purported, on the basis of a 0.93 correlation coefficient, to give essentially the same information (7).

Although the CDAI has been used in clinical trials involving children, it has limited applicability to children and adolescents especially as it does not take growth and development into account.

### Initial Pediatric Severity Scores

The first activity scoring systems for children with CD were developed in the 1970's. Whittington and colleagues (8) reported the evaluation of disease severity in a series of 16 adolescents. The scoring system included patient symptoms, physical examination findings and the results of two blood markers: erythrocyte sedimentation rate (ESR) and albumin. Severity was assessed on a six-point scale, where 1 was asymptomatic with normal bloods and 6 was incapacitating symptoms. This scale was used in the description of a small group of patients to delineate response to medical therapies over time. It did not include assessment of growth, was not formally evaluated or validated and has not been reported in any other patient groups.

Two years later, Lloyd-Still and Green (9) reported their scoring system for children with IBD. Included within this score were growth data, endoscopic findings, and radiological findings. Severity correlated with serum albumin levels. While the score was reported to be easy to use and reliable between reporters, it required invasive investigations and has not been used widely.

### Pediatric Crohn Disease Activity Index (PCDAI)

The Pediatric Crohn Disease Activity Index (PCDAI) was developed in 1990 with the input of 30 pediatric gastroenterologists from 12 North American centers with the goal to be a tool that could be used easily and reproducibly for longitudinal assessment of disease activity in multi-center studies (10). The tool is comprised of 11 items (symptoms, physical examination, growth and selected serum inflammatory markers) completed by a physician with scores ranging from 0 to 100, with higher scores indicating worse disease activity. The development and validation of the tool included collection of relevant data from 131 children with CD. Physicians completed a modified HBI and provided Physician Global Assessment (PGA) scores for each of the patients.

The analysis of the data arising from these assessments showed that the PCDAI correlated well with HBI and PGA scores. It also provided delineation between remission and degrees of severity. The key benefits of the PCDAI over the HBI was the inclusion of growth parameters and objective markers of inflammation, whilst providing a clear scoring range.

Hyams et al. (11) in a study published in 2005, aimed to evaluate the responsiveness of the PCDAI to changes in the status of patients after therapeutic interventions. Data were derived from a prospective database of newly diagnosed children with IBD established in 2002 at 18 pediatric gastroenterology centers in the United States and Canada. Data were examined from the 95 patients with either 30 day or 3 months follow up data to determine the magnitude of PCDAI changes noted following initial therapeutic intervention. A PCDAI score of 10 appeared to give the best balance between sensitivity and specificity in distinguishing between inactive and mild disease. Large average decreases in PCDAI were observed for those patients with moderate/severe disease by PGA at diagnosis who improved to either inactive or mild disease. A PCDAI score of >30 showed relatively good sensitivity (0.71) while also providing acceptable specificity (0.83) discriminating moderate/severe from mild disease. Clinical response (moderate/severe disease improving to mild/inactive) was best reflected by a decrease in PCDAI of >12.5 points. Subgroup analysis for patients with severe disease at diagnosis showed a higher mean PCDAI decrease (11).

Otley et al. (12) subsequently compared the feasibility, validity, and responsiveness of the PCDAI compared to the CDAI in the assessment of disease activity in children with CD. This study demonstrated that the PCDAI was superior to the CDAI in discriminating between levels of disease activity.

The short term responsive of the PCDAI was demonstrated by Kundal et al. (13). In this evaluation, PCDAI scores were contrasted to PGA scores. A change of 12.5 points in the PCDAI was shown to clearly represent a change in physician-assessed severity. The authors concluded that the tool was valid to consider in clinical trials.

Loonen et al. (14) subsequently analyzed the value of six “criticized” items to the discriminative properties of the PCDAI. These items included three laboratory items (hematocrit, erythrocyte sedimentation rate, and albumin) and three physical items (height, perirectal disease, and extraintestinal manifestations). They showed that a clinical index, later called the abbreviated PCDAI (abbrPCDAI), consisting of three history items (abdominal pain, number of liquid stools, and general well-being) and three physical examination items (weight loss, abdominal examination, and perirectal disease) has an accuracy equal to the standard PCDAI in distinguishing children with disease in remission from those with a relapse. Shepanski et al. (15) further examined the abbrPCDAI and also found that it predicted disease activity as well as the full PCDAI.

Leach et al. (16) re-evaluated the PCDAI score in a different fashion by excluding subjective elements and adding C-reactive protein (CRP) levels as a fourth objective laboratory marker. This modified version of the PCDAI (mod-PCDAI) performed well in an assessment against PGA and fecal calprotectin values, but the initial evaluation was undertaken in just one center and included a relatively small cohort of children.

Kappelman et al. (17) presented a short version of the PCDAI (shPCDAI), excluding items with a low frequency of completion in a patient registry. The difference between the shPCDAI from the abbrPCDAI is that the extraintestinal manifestation item replaced the perianal item, and that new weightings were mathematically assigned to each item by multivariate modeling, reflecting their relative importance to physician global assessment of disease activity.

More recently, Turner et al. (18) examined a mathematically weighted version of PCDAI (wPCDAI) based on reducing less important and unnecessary items of the PCDAI according to the judgement of a group of experts. The authors then used large prospectively collected data sets to mathematically weigh the individual items of the PCDAI. They showed that the wPCDAI discriminated better between the disease activity categories and therefore yielded a more feasible, reliable, valid, and responsive index.

There is very limited data correlating the PCDAI and the other versions of this index with mucosal inflammation (3). Turner et al. (3) aimed to compare four PCDAI versions head-to-head with endoscopic degree of inflammation as measured by the Simple Endoscopic Score for Crohn's Disease (SES-CD), fecal calprotectin, ESR, and C-reactive protein (CRP) and to explore cut-off values associated with mucosal healing. They used the prospectively collected data from the ImageKids study that included 100 children with CD undergoing colonoscopy (19) and 222 children from the Growth Relapse and Outcomes with Therapy study for which 145 children had calprotectin data 12 weeks after diagnosis (20). Both wPCDAI and PCDAI were found to be superior to the shorter versions when comparing the blood tests. All versions had poor correlation with calprotectin, and only the wPCDAI reached significance (3).

Griffiths et al. (21) subsequently reviewed the utility of activity indices and end points for clinical trials in children with CD. This was the first attempt to critically review the performance characteristics of existing measures of disease activity scores as well as health related quality of life measures and factors that complicate the evaluation of linear growth. They summarized the recommendations based on Levels 1 and 2 evidence for using activity indices for acute treatment trials and for maintenance of remission/prevention of postoperative recurrence trials and long-term registries. They suggested to measure both the PCDAI and CDAI (with pediatric modification) along with physician and patient/parent global assessment of disease activity and of change to facilitate confirmation of appropriate cut scores and definitions of response/loss of response for both.

## Ulcerative Colitis Disease Activity Scores

### Mayo Score

The most widely used UC activity index in adult clinical trials is the Mayo score (22). This score, which was introduced in 1987, comprises three clinical parameters (each of which is assigned a score from 0 to 3) and an endoscopic score.

Subsequently, a simplified clinical or partial Mayo Score has been developed (23, 24). This tool uses the three non-invasive components of the full Mayo Score (stool frequency, rectal bleeding and physician's global assessment) and excludes the endoscopic findings. Consequently, the maximum score is reduced from 12 to 9 points. This simplified index maintains a good relationship with the full Mayo Score in identifying clinical response as perceived by patients (23, 24).

### Pediatric Ulcerative Colitis Activity Index (PUCAI)

Since colonoscopy is less used in children to assess disease activity, Turner et al. (25) developed a non-invasive activity index for pediatric UC (PUCAI score). Item selection was performed judgmentally using a Delphi group of 36 pediatric IBD experts. Item weighting was performed by regression modeling using a prospective cohort of 157 children with UC. Validation was then assessed with a separate prospective cohort of 48 children with UC undergoing full colonoscopy. Responsiveness was also evaluated at a follow-up visit of 75 children using effect size statistics and diagnostic utility approaches. An initial 41 item list was then reduced to 11 by rank order. Two physicians completed the PUCAI on each of the patients in the weighting cohort.

Six clinical items were found to be significant in the regression analysis (abdominal pain, rectal bleeding, stool consistency, number of stools per 24 h, nocturnal bowel movement and activity level). The addition of laboratory items or an endoscopic appearance item did not improve the performance of the PUCAI.

In the validation cohort, the PUCAI was highly correlated with the PGA (*r* = 0.91, *P* < 0.001), Mayo score (*r* = 0.95, *P* < 0.001), and colonoscopic appearance (*r* = 0.77, *P* < 0.001). Excellent responsiveness was also found at repeated visits.

In another study, Turner et al. (26) performed a prospective study to compare all non-invasive disease activity indices in patients with UC and to identify cutoff scores that correspond to remission and response. The study included 86 adults with UC (52% males, mean age 37.6 ± 13.7 years). The following scores were used: partial Mayo score, Rachmilewitz, Lichtiger, Seo, PUCAI, Partial Powell-Tuck, Endoscopic-Clinical Correlation, Beattie, and Walmsley. Physician and patient global assessments, colonoscopic scores, blood test data, and the full Mayo scores were used to assess construct and discriminative validity. A follow-up evaluation of 61 patients was used to assess test-retest reliability and responsiveness.

The Walmsley index and PUCAI were best in assessing disease activity, determined by all four clinimetric properties. In assessing validity, the mean correlation coefficients for the five included constructs were *r* = 0.80 and *r* = 0.79 for the Walmsley and PUCAI, respectively (*P* < 0.001 for each). The partial Mayo score accurately determined disease activity in three of the four clinimetric properties; the Rachmilewitz index accurately assessed patients in two of the properties. Cutoff scores that defined combined clinical-endoscopic remission and response were determined using receiver operating characteristic curve analyses for all instruments. They concluded that the Walmsley index and PUCAI are valid, reliable and responsive non-invasive measures to assess disease activity in adults with UC. The authors further suggested that use of these indices might permit less frequent endoscopic assessment in patients with UC—both in research and in clinical practice (26).

Kerur et al. (27) investigated whether the PUCAI score correlates with mucosal inflammation and endoscopic assessment of disease activity (Mayo endoscopic score). They reviewed charts from patients with UC who had undergone colonoscopy over 3 years. Clinical assessment of disease severity within 35 days (either before or after) the colonoscopy were included. Patients were excluded if they had significant therapeutic interventions (such as the start of corticosteroids or immunosuppressive agents) between the colonoscopy and the clinical assessment. The Mayo endoscopic score of the rectum and sigmoid were completed by two gastroenterologists. Inter-observer variability in Mayo score was assessed. They identified 99 patients (53% female, 74% pancolitis) that met inclusion criteria. The indications for colonoscopy included ongoing disease activity (62%), consideration of medication change (10%), assessment of medication efficacy (14%), and cancer screening (14%). Based on PUCAI scores, 33% of patients were in remission, 39% had mild disease, 23% had moderate disease, and 4% had severe disease. They concluded that endoscopic disease severity generally correlates with clinical disease severity as measured by PUCAI score. However, children with colitis may have significant variation in their reported clinical symptoms and therefore suggested consideration of endoscopic assessment in that setting (27).

In a recent study, Ricciuto et al. (28) showed that children with primary sclerosing cholangitis and IBD (PSC-IBD) in clinical remission, based on PUCAI scores, have a significantly higher risk of active endoscopic and histologic disease than children with UC without PSC. These data indicated that PUCAI scores underperform in the setting of UC with PSC.

## Patient vs. Physician Disease Severity Assessment

Clinical IBD indices completed by adult patients typically do not correspond well with physicians completed indices (29). Patient-reported outcome measures (PROMs) have increasingly become an important part of disease assessment in adults. Louis et al. (30) employed a three-step approach to develop an IBD descriptive framework. They started with a literature review to identify a broad list of attributes, and subsequently arranged focus group meetings of patients and clinicians to assess the relevance of the attributes. Two rounds of voting were undertaken to name and define each attribute.

A total of 10 attributes were identified: abdominal pain, other disease-related pain, bowel urgency, fatigue, risk of cancer and serious infections within the next 10 years, risk of mild to moderate complications, aesthetic complications related to treatment, emotional status, sexual life, and social life and relationships. They suggested that this descriptive framework should be considered by physicians when discussing IBD treatment options with their patients.

Schreiber et al. (31) examined perceptions of symptoms and their management between adult patients with UC and healthcare professionals (HCPs). Structured, cross-sectional, Web-based questionnaires designed to assess a variety of disease indices were completed by patients with UC and HCPs from Canada and several European countries. They found that patient self-reported classification of disease severity revealed generally greater severity (mild, 32%; moderate, 53%) compared with physician and nurse estimates (mild, 52 and 49%; moderate, 34 and 37%, respectively). Patients reported an average of 5.5 flares (self-defined) occurred over the past year, compared with 3.4 and 3.8 flares per year estimated by physicians and nurses. About half of patients (47%) defined remission as experiencing no symptoms; by comparison, 62–63% of HCPs defined remission as requiring the complete absence of symptoms.

The IBD Disability Index (IBD-DI) is a physician-administered tool that evaluates the functional status of patients with IBD (32, 33). The IBD-DI was developed using a formal consensus process and was subsequently validated (32, 33). Based on this index Ghosh et al. (34) developed the IBD Disk, A Visual Self-administered Tool for Assessing Disability in Inflammatory Bowel Diseases. After four rounds of voting, the following 10 items were agreed upon for inclusion in the IBD Disk: abdominal pain, body image, education and work, emotions, energy, interpersonal interactions, joint pain, regulating defecation, sexual functions, and sleep. All elements, except sexual functions, were included in the validated IBD Disability Index. Recently, this tool was validated by Le Berre et al. (35). They included 447 patients in the analysis at baseline and 265 at follow-up (71% Crohn's disease, 28% ulcerative colitis). Their patients with IBD responded twice to both IBD-Disk and IBD-DI at 3–12 months intervals. There was a good correlation between IBD-Disk and IBD-DI scores (*r* = 0.75, *p* < 0.001). Reproducibility was excellent (intra-class correlation coefficient = 0.90), as well as internal consistency (Cronbach's α = 0.89).

Diederen et al. (29) investigated the agreement between patient- and physician-based clinical indices in children and adolescents with a previous diagnosis of IBD. This was a cross-sectional study that prospectively enrolled children aged 8–18 years with IBD. Patients with CD completed a patient-based shPCDAI while those with UC or IBDU completed the PUCAI. Physicians completed the original physician-based shPCDAI or PUCAI. Agreement was calculated with linear weighted kappa.

In total, 154 pairs of clinical indices were collected: 89 pairs of shPCDAI scores (median age at assessment 15.6 years, 61% male) and 55 paired PUCAI scores (median age at assessment 14.0 years, 44% male). The shPCDAI disease activity category only fairly agreed between patient- and physician-based indices [kappa: 0.40 (95% confidence interval 0.24–0.55), *P* < 0.001], with perfect agreement in 58% of pairs. In the majority of disagreement (81%), patients scored in a higher shPCDAI disease activity category. The PUCAI disease activity category substantially agreed between patient- and physician-based indices [kappa: 0.64 (95% confidence interval 0.45–0.83), *P* < 0.001], with perfect agreement in 78% of pairs. In the majority of disagreement (75%), patients scored in a higher PUCAI disease activity category. The authors concluded that patient- and physician-based shPCDAI and PUCAI do not always agree, particularly the shPCDAI, and therefore, should not be interpreted equivalently in management and research on children and adolescents with IBD (29).

More recently, Vernon-Roberts et al. (36) developed a new self-report tool for children. The IBD-NOW tool was designed to use picture and text scales to better represent current symptoms from the child's perspective. The new tool was assessed in 100 children (88 with CD) with comparison to physician completed PCDAI or PUCAI scores. The IBD-NOW performed well with an overall Cronbach alpha of 0.74. Although this tool appears to be a promising way for children present their current symptoms, it is yet to be assessed in any other locations. In addition, the development cohort predominantly included children with CD: further evaluation in larger groups of children with UC is also required.

## Endoscopic Severity Scores

Numerous systems to grade endoscopic severity have been developed and assessed over time, for both CD and UC. The range of scores have been reviewed in several reports (37–39). Some key and most widely accepted scores are the SES-CD, CDEIS, Rutgeerts, and Mayo.

### Crohn's Disease Endoscopic Index of Severity (CDEIS)

The CDEIS assesses the endoscopic appearance in four colonic segments and the terminal ileum (40). The surface is assessed in terms of ulceration and stenosis and the extent of these changes, with a score ranging from 0 to the maximum (worst) score of 44. This scoring system has been utilized in clinical trial settings, but there is more discrepancy about the optimal cut-off to indicate endoscopic response and remission.

### Simple Endoscopic Score for CD (SES-CD)

The SES-CD was designed to be simpler and quicker to complete than the CDEIS (41). It is scored from 0 to 60, with consideration of presence of ulceration, extent of ulceration, extent of endoscopic involvement, and stenosis within each of five segments of the ileocolon. A >50% reduction in SES-CD score is considered endoscopic response, while a score of <2 is considered to indicate endoscopic remission (42).

Ledder et al. (43) have recently developed a specific SES-CD for the upper gut in children with CD (UGI-SES-CD). Scores were completed for the segments of the upper gut in 202 children with fully characterized CD. These scores at diagnosis were associated more severe disease activity (such as higher wPCDAI scores) but not with the subsequent course of disease.

In a related study, Church et al. (44) demonstrated that the findings present in the upper gut of 188 children were not able to be reliably detected by MRE. The authors concluded that MRE could not be used to replace the need for upper gastrointestinal endoscopy in children with suspected CD.

### Mucosal Inflammation Non-invasive Index (MINI) for Pediatric Crohn's Disease

Cozijnsen et al. (45) aimed to develop and validate a non-invasive index (the Mucosal Inflammation Non-invasive Index *-* MINI) to assess mucosal inflammation in children with CD. They collected data from the ImageKids study and investigated the association of PCDAI items and laboratory test results with the simple endoscopic score for CD (SESCD). These data have been used in a blended mathematical judgmental clinimetric approach to develop a weighted categorized index to identify children with CD who have MH. The index has been validated using data from three independent patient cohorts. The derivation and validation cohorts included 154 and 168 children, respectively (age 14.1 ± 2.5 years and 14.2 ± 3.9 years), of whom 16 and 36% had MH (defined as SESCD <3). The authors showed in multivariable models that the stooling item of the PCDAI, ESR, and fecal calprotectin level were associated with SESCD. CRP level has been also added to the score. MINI scores below 8 identified children with MH with 88% sensitivity and 85% specificity in the derivation cohort and with 84% sensitivity and 87% specificity in the validation cohorts. Ninety percent of the patients in the validation cohort with scores of eight or more had active mucosal inflammation. Scores below six increase the positive predictive value to 86%. The added benefit of MINI over measurement of fecal calprotectin was small but significant, especially for patients with concentrations of fecal calprotectin between 100 and 599 mg/g (45).

### Rutgeerts Score

The Rutgeerts score is focused upon the grading of endoscopic recurrence after surgical resection (46). A score of i0 (normal anastomosis) or i1 (<5 aphthous ulcers) 6–12 months post-operatively predicts a low risk of clinical recurrence in the subsequent 2–3 years. In contrast, a score of i3 (diffuse inflammation and ulceration) or i4 (stenosis or nodules) is associated with almost certain risk of subsequent clinical recurrence. Whilst not formally validated in children, this score has been used in children as well as adults in clinical and research trial settings.

### Mayo

The Mayo score evaluates the endoscopic severity using a four-point scale with zero being normal and three indicating the presence of ulceration and spontaneous bleeding (22). Although not formally evaluated in adults or children, this score is simple, reproducible and used extensively in clinical trial settings. The modified Mayo score extends this score by assessing changes within each of five colonic segments with an averaging of involvement to reach the final score (47). This score is shown to correlate with other markers of disease activity.

## Magnetic Resonance Enterography Severity Scores

Magnetic resonance enterography (MRE) is now well-established as a cross-sectional imaging modality to ascertain the presence or extent of small bowel disease in individuals with CD and to delineate disease-related complications such as small bowel stricture. MRE has significant benefits over other cross-sectional imaging methods, such as computed tomography, due to avoidance of ionizing radiation, especially in children and adolescents. Several tools have been developed to provide objective assessment of MRE findings, with most assessments focusing on adult patients rather than children.

### Crohn's Disease MRI Index (CDMI)

The CDMI, also known as the London index or Crohn's disease activity score, was developed in 2012 (48). Various specific MRI findings were scored from 0 to 3 and compared to a pathological severity score. The score utilizes mural thickness and T2 signal in the following equation: 1.79 + 1.34 mural thickness + 0.94 mural T2 score. This score provided sensitivity of 0.81 and specificity of 0.70 for the detection of acute inflammation.

### Magnetic Resonance Enterography Global Score (MEGS)

This score, developed in 2014, modified the CMI by the inclusion of additional features (length of involved gut, loss of colonic haustra and presence of features such as fistula or lymphadenopathy) (49). The score involves dividing the small and large intestine into nine sections, with summation of scores for each section to provide a total score. MEGS was validated in patients with CD and compared favorably to other markers such as fecal calprotectin.

Recently, MEGS was evaluated in 52 Chinese children with CD and compared directly to SES-CD scores in each child (50). MEGS scores correlated with endoscopic scores (*r* = 0.70, *p* < 0.001). Further, MEGS scores predicted disease activity with sensitivity of 88% and specificity of 75%.

### Magnetic Resonance Index of Activity (MaRIA)

The MaRIA score is derived from assessment of mural thickness, contrast enhancement, oedema and ulceration using a specific formula (51). The total score is derived from the summation of scores for each section of the gut.

In a recent study Minordi et al. (52) evaluated the findings from repeated MRE in 46 individuals with CD before and after medical therapy. MaRIA scores reflected clinical responses (judged using Harvey Bradshaw indices).

The MaRIA has been evaluated in one recent pediatric study (53). This sub-study of the ImageKids study (designed to develop and evaluate pediatric inflammatory and damage MRE scores) included 237 children with CD. Most (83%) had completed ileocolonoscopy—the remainder did not have ileal intubation. Overall MaRIA scores agreed strongly (75%) with the ileal endoscopic scores (SES-CD). The agreement between the colonic segment endoscopic scores and MaRIA scores ranged between 68 and 85%. The authors developed a model to be able to predict the ileal SES-CD scores in the subset of children who did not have ileal intubation.

### Diffusion-Weighted Imaging (DWI)-MaRIA Score

This imaging method is performed using a T2-weighted MRI sequence with fat suppression and addition of a diffusion gradient. This method was employed in the development of the DWI-MaRIA score (also known as the Clermont score) (54). The DWI-MaRIA score includes apparent diffusion co-efficient, mural thickening, ulceration, and oedema. A score >8.4 was very predictive of ileal activity, while a score >12.5 was indicative of severe activity.

The DWI-MaRIA score was recently contrasted to MaRIA and the CDMI tools in 98 patients with newly diagnosed CD assessed with MRI and ileocolonoscopy with biopsies (55). Each of the MRI scoring systems provided comparable information in reference to endoscopic and histological references. Again this tool has not yet been evaluated in children with IBD.

### Simplified MaRIA (sMaRIA)

The simplified version of the MaRIA tool (sMaRIA) was recently developed and validated by Spanish investigators (56). Four factors (ulceration, fat stranding, oedema, and wall thickening) were utilized in this scoring system, which was evaluated in a cohort of patients with CD who underwent endoscopic and MRE assessment before and after therapy. The sMaRIA was highly correlated with endoscopic severity (CDEIS) scores (*r* = 0.83). A Portuguese group have subsequently independently validated the sMaRIA in 84 patients with CD (57). The results arising indicated that sMaRIA correlated strongly with endoscopic severity (*r* = 0.95) and with levels of fecal calprotectin (*r* = 0.91).

### Pediatric Inflammatory Crohn's MRE Index (PICMI)

The PICMI score was developed and validated in Toronto, Canada (58). The imaging findings of 48 children with CD were evaluated. The MRE signs correlated with wPCDAI scores, and several findings (including wall enhancement and thickening) were more strongly associated with inflammation. This score has not yet been evaluated further or in other settings.

## Conclusions

A number of scoring tools have been developed for the assessment of disease status in individuals with IBD. These focus on clinical, endoscopic, or radiological assessment of disease severity or activity. Several of the available tools have been evaluated or validated in children with IBD and take into account the patterns and impacts of IBD in this age group.

The PCDAI versions and PUCAI scoring are widely used in clinical practice, decision making algorithms in CD treatment and acute severe UC, as well as clinical research throughout the world, with available clinical calculators and smart phones applications.

Appropriately validated tools to assess disease status are relevant to the diagnosis of IBD and also to follow up, delineation of response to therapy and ascertainment of disease relapse. Furthermore, standardized tools permit enhanced communication between physicians at clinical and research levels. The development and validation of pediatric-focused tools is essential in enabling optimal care and outcomes for children with IBD.

## Data Availability Statement

The original contributions presented in the study are included in the article/supplementary material, further inquiries can be directed to the corresponding author/s.

## Author Contributions

All authors did the literature search, wrote the manuscript, and approved the final version.

## Conflict of Interest

The authors declare that the research was conducted in the absence of any commercial or financial relationships that could be construed as a potential conflict of interest.
